# Association between clinical factors and orofacial dyskinesias in anti‐N‐methyl‐D‐aspartate receptor encephalitis

**DOI:** 10.1002/brb3.2638

**Published:** 2022-05-27

**Authors:** Hailun Hang, Liuyu Lin, Danhui Li, Jin Li, Jingping Shi, Jie Lu

**Affiliations:** ^1^ Department of Neurology The Affiliated Brain Hospital of Nanjing Medical University Nanjing China; ^2^ Department of Radiology Beijing Chao‐Yang Hospital Capital Medical University Beijing China

**Keywords:** anti‐NMDAR, autoimmune encephalitis, movement disorders, orofacial dyskinesias

## Abstract

**Background and Purpose:**

We aimed to determine whether demographic information, clinical characteristics, laboratory tests, and imaging features are associated with orofacial dyskinesias (OFLD) in patients with anti‐N‐methyl‐D‐aspartate receptor (NMDAR) encephalitis.

**Methods:**

In this retrospective study, patients who were diagnosed with anti‐NMDAR encephalitis were enrolled. All patients’ factors, including demographic information, clinical characteristics, laboratory tests, and imaging features, were obtained at the time of hospitalization. The neurological function was assessed using the modified Rankin scale (mRS). Univariate and multivariate logistic regressions were used to examine the associations between clinical factors and OFLD.

**Results:**

In total, 119 patients (median age: 28.0 [19.0–41.0] years; 67 females) were recruited. Of 119 patients, 44 (37.0%) had OFLD. OFLD was associated with increased mRS at admission, serum sodium, lumbar puncture pressure, female biologic sex, fever, psychiatric symptoms, seizures, impaired consciousness, autonomic dysfunction, and central hypoventilation in univariate logistic regression, respectively. Multivariate regression analysis revealed that female biologic sex (odds ratios [OR], 4.73; 95% confidence interval [CI], 1.27–17.64; *p* = .021), increased mRS at admission (OR, 2.09; 95% CI, 1.18–3.71; *p* = .011), psychiatric symptoms (OR, 7.27; 95% CI, 1.20–43.91; *p* = .031), and seizures (OR, 5.11; 95% CI, 1.22–21.43; *p* = .026) were associated with OFLD, after adjusting for confounding factors.

**Conclusions:**

Our analysis suggests that the following clinical factors are associated with OFLD: female biologic sex, increased mRS at admission, psychiatric symptoms, and seizures.

## Graphical abstract

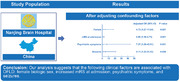

## 1 INTRODUCTION

Anti‐N‐methyl‐D‐aspartate receptor (NMDAR) encephalitis, i.e., the presence of cerebrospinal fluid (CSF) antibodies against the GluN1 subunit of the NMDAR, is an immune‐mediated disease and the most common autoimmune encephalitis (AE) (Dalmau & Graus, 2018; Dalmau et al., 2011). The most frequent manifestations of anti‐NMDAR encephalitis include psychiatric symptoms, movement disorders (MDs), cognitive decline, epilepsy, central hypoventilation, and autonomic nervous dysfunction (Dalmau et al., 2008; Titulaer et al., 2013). It is well established that MDs are a frequent symptom in anti‐NMDAR encephalitis and may be observed from the initial stages of disease to the comatose stage (Dalmau et al., 2011). MDs occur either in isolation or, more commonly, as a prominent feature of the condition and may appear as the first sign in children (Udani et al., 2015). Available evidence suggests that orofacial dyskinesias (OFLD) is a distinctive feature of MDs in anti‐NMDAR encephalitis, which usually appears during the phase of unresponsiveness and catatonia (Dash & Pandey, 2019; Duan et al., 2016). Moreover, severe OFLD may lead to oral and lingual injuries and damaged teeth (Dalmau et al., 2008; Di Luca & Margolesky, 2019).

Previous studies have reported OFLD as a possible indicator of illness progression, which may assist in clinical assessment and treatment planning (Duan et al., 2016; Epstein & Difazio, 2007; Morales‐Briceño & Fung, 2017; Zheng et al., 2018). Moreover, current literature suggests that co‐morbid MDs are more common in biologically female individuals diagnosed with anti‐NMDAR encephalitis (Varley et al., 2019). However, demographic differences and clinical characteristics of OFLD in anti‐NMDAR encephalitis, such as age, sex, and clinical symptoms, have not been well investigated. Therefore, in this study, we aimed to determine the associations between clinical factors and OFLD were present in patients with anti‐NMDAR encephalitis.

## 2 METHODS

### 2.1 Study population

In this retrospective study, we investigated the presence of NMDAR antibodies in the both serum and CSF samples of patients at the Affiliated Brain Hospital of Nanjing Medical University from July 2014 to December 2020. We recruited patients diagnosed with anti‐NMDAR encephalitis based on the previously published criteria (Dalmau et al., 2008; Graus et al., 2016). The exclusion criteria for the study were as follows: (1) no CSF examination or incomplete clinical data from the period of hospitalization; (2) central nervous system infection caused by other specific intracranial pathogens (such as herpes simplex virus 1 and 2, and herpes zoster virus); (3) an immunosuppressed state (including long‐term immunosuppressive therapy due to chemotherapy or organ transplantation); and (4) thyroid disease, a recent history of thyroid hormone replacement, or a lack of test results on thyroid function and antibodies. This study was approved by the Ethics Committee of the Affiliated Brain Hospital of Nanjing Medical University. All the patients provided written consent forms.

### 2.2 Demographic information and clinical characteristics

The demographic information of all the patients, including age and sex, was collected from the electronic medical record system. A neurologist analyzed clinical characteristics at hospitalization. The presence or absence of headaches, fever, cognitive decline, psychiatric symptoms, MDs, seizures, impaired consciousness, autonomic dysfunction, speech disturbance, central hypoventilation, and sleep disturbance was evaluated, respectively. The category and diagnosis of MDs that included OFLD, tremors, dystonias, stereotypies, chorea, myoclonus, catatonia, and parkinsonism were assessed according to previous studies (Damato et al., 2018; Varley et al., 2019). Moreover, the time from the onset to admission (TOA), time from the onset to diagnosis (TOD), and length of stay were recorded for all the patients. The neurological function of all the patients at admission was independently evaluated by two physicians with consensus using the modified Rankin scale (mRS) (van Swieten et al., 1988).

### 2.3 Laboratory tests, electroencephalography, and imaging examination

All the patients underwent laboratory tests, including standard biochemistry, AE‐related antibodies (Dalmau & Graus, 2018; Hang et al., 2020), thyroid function, rheumatic indicators, syphilis, tumor biomarkers, autoantibodies, and other laboratory tests. The patients also underwent both CSF and serum examinations. The blood and CSF AE‐related antibodies were detected by indirect immunofluorescence testing based on cell‐based assay as previously published (Dalmau et al., 2017). The lumbar puncture pressure (LPP), CSF cell count, and CSF protein were measured. All the patients underwent brain magnetic resonance imaging (MRI), routine electroencephalography (EEG), and abdominal ultrasonography examinations. Thyroid function abnormalities included continuous thyroid function test measurements (abnormal concentrations of thyrotropin and FT4), or thyroid peroxidase antibody positivity, or thyroglobulin antibody positivity. Abnormal EEG was defined as extreme delta brush, focal or diffuse slow or disorganized activity, or epileptic activity (Graus et al., 2016). The T2/T2‐fluid attenuated inversion recovery hyperintensities in the unilateral or bilateral limbic system, or other areas, or multiple cortical or basal ganglia were considered as abnormal brain MRI (Graus et al., 2016).

### 2.4 Statistical analysis

The continuous variables were described as the mean ± standard deviation (SD) or median and interquartile range (IQR); all the categorical variables were expressed as frequency (percentage). The independent *t*‐test and Mann–Whitney *U* test were used to compare differences between continuous variables, and the differences of categorical variables were compared with chi‐squared or Fisher's exact test. Univariate and multivariate logistic regressions were used to determine the odds ratios (OR) and 95% confidence intervals (CI) of demographic or clinical factors in discriminating the presence of OFLD. Variables showing a univariate relationship with OFLD (*p* < .1) were entered into a multivariate logistic analysis model. Multi‐collinearity of variables was diagnosed by tolerance of <0.1 or variance inflation factor of >10. The *p*‐value < .05 was considered statistically significant. The statistical analyses were conducted using SPSS 19 (IBM Corporation, Armonk, New York, USA).

## 3 RESULTS

### 3.1 Demographic information, clinical characteristics, laboratory tests, and imaging features

Of the 140 enrolled patients, 21 were excluded, including 12 with incomplete clinical information and 9 cases positive for multi‐antibodies types in the CSF and serum. Of the remaining 119 patients, 52 (43.7%) were male and 67 (56.3%) were female, aged 28.0 (IQR: 19.0–41.0). In our sample of 119 patients, specific symptoms were reported which include the following: headaches (N = 42, 35.3%), fever (N = 69, 58.0%), cognitive decline (N = 47, 39.5%), psychiatric symptoms (N = 91, 76.5%), seizures (N = 84, 70.6%), impaired consciousness (N = 33, 27.7%), autonomic dysfunction (N = 9, 7.6%), speech disturbance (N = 33, 27.7%), central hypoventilation (N = 16, 13.4%), and sleep disturbance (N = 24, 20.2%).

In total, the prevalence rates of tumor biomarkers, rheumatic indicators, autoantibodies, abnormal thyroid function, teratoma, abnormal EEG, and abnormal brain MRI were 34.5%, 63.0%, 17.6%, 62.2%, 2.5%, 84.9%, and 19.3%, respectively.

### 3.2 Differences in demographic information, clinical characteristics, laboratory tests, and imaging features between the OFLD and non‐OFLD groups

**FIGURE 1:**
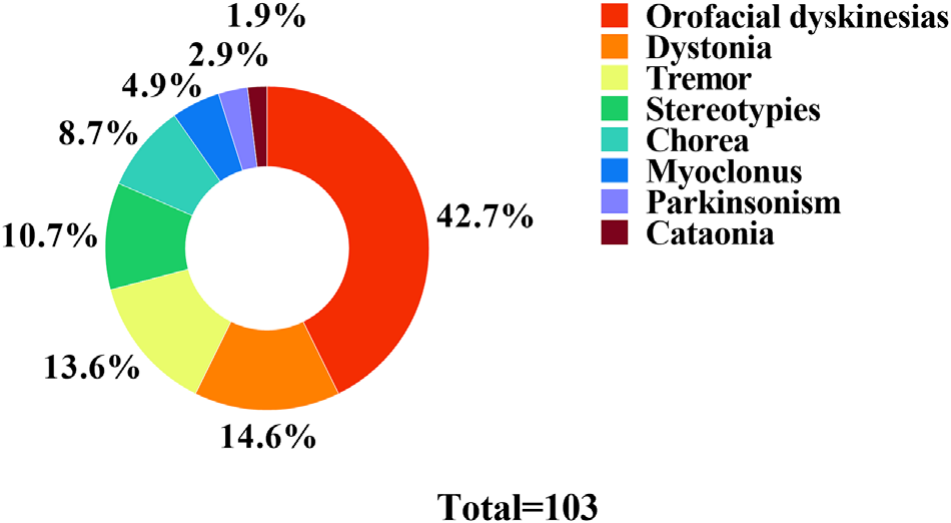
The prevalence and category of movement disorders (MDs) in 119 patients with anti‐N‐methyl‐D‐aspartate receptor (NMDAR) encephalitis. Orofacial dyskinesias were the most common in MDs. Other MDs, such as dystonia, tremor, and stereotypies, were more frequent in anti‐NMDAR encephalitis

There were 103 MD cases in the present study, of which 44 (42.7%) were confirmed as OLFD based on the presence of the characteristic movements of chewing, jaw opening, sucking, and lip‐smacking. Remaining MDs included dystonia (N = 15, 14.6%), tremor (N = 14, 13.6%), stereotypies (N = 11, 10.7%), chorea (N = 9, 8.7%), myoclonus (N = 5, 4.9%), parkinsonism (N = 3, 2.9%), and catatonia (N = 2, 1.9%). The prevalence and category of all MDs are detailed in Figure 1.

In OFLD group, the patients exhibited higher mRS at admission (4.0 [IQR: 3.0–5.0] vs. 3.0 [IQR: 2.0–4.0], *p *< .001) and serum sodium (142.0 [IQR: 139.2–143.9] 10^9^/L vs. 140.4 [IQR: 137.4–143.0] 10^9^/L, *p* = .026). Female biologic sex (79.5% vs. 42.7%, *p *< .001), fever (81.8% vs. 44.0%, *p *< .001), psychiatric symptoms (90.9% vs. 68.0%, *p* = .004), seizures (84.1% vs. 62.7%, *p* = .013), impaired consciousness (40.9% vs. 20.0%, *p *= .014), autonomic dysfunction (15.9% vs. 2.7%, *p *= .023), and central hypoventilation (25.0% vs. 6.7%, *p *= .005) were observed at higher frequency in OFLD group than those without OFLD. No significant differences were found in other clinical characteristics, laboratory tests, imaging features, and age between the two groups (all *p* ≥ .05). Table 1 presents the results of comparisons between the two groups.

**TABLE 1:** The comparison of clinical factors between two groups

	Mean ± SD, median (IQR), or N (%)		
	OFLD group (*n* = 44)	Non‐OFLD group (*n* = 75)	*p*‐Value
**Demographic information and clinical characteristics**			
Age, years	25.0 (19.0–31.8)	30.0 (21.0–47.0)	.085
Sex, female	35 (79.5)	32 (42.7)	<.001
Length of stay, days	31.0 (21.0–42.5)	24.0 (19.0–34.0)	.095
TOA (range), days	15.0 (8.3–27.8)	18.0 (7.0–30.0)	.733
TOD (range), days	22.0 (12.0–31.0)	22.0 (13.5–34.0)	.448
mRS at admission	4.0 (3.0–5.0)	3.0 (2.0–4.0)	<.001
Headache	14 (31.8)	28 (37.3)	.543
Fever	36 (81.8)	33 (44.0)	<.001
Cognitive decline	16 (36.4)	31 (41.3)	.699
Psychiatric symptoms	40 (90.9)	51 (68.0)	.004
Seizures	37 (84.1)	47 (62.7)	.013
Impaired consciousness	18 (40.9)	15 (20.0)	.014
Autonomic dysfunction	7 (15.9)	2 (2.7)	.023
Speech disturbance	15 (34.1)	18 (24.0)	.235
Central hypoventilation	11 (25.0)	5 (6.7)	.005
Sleep disturbance	13 (29.5)	11 (14.7)	.051
**Laboratory tests, EEG and brain MRI**			
Blood leukocyte, 10^9^/L	9.67 (7.86–10.94)	8.73 (7.17–12.05)	.743
Serum sodium, 10^9^/L	142.0 (139.2–143.9)	140.4 (137.4–143.0)	.026
Serum potassium, 10^9^/L	3.73 ± 0.34	3.78 ± 0.39	.516
Serum calcium, 10^9^/L	2.28 ± 0.13	2.25 ± 0.28	.515
LPP, mmHg	160.0 (101.3–227.5)	127.5 (89.5–175.0)	.059
CSF cell count, per/μl	18.0 (5.0–63.0)	8.0 (4.0–30.5)	.074
CSF protein, g/L	0.46 (0.32–0.55)	0.46 (0.36–0.66)	.369
Tumor biomarkers	17 (38.6)	24 (32.0)	.829
Rheumatic indicators	33 (75.0)	42 (56.0)	.775
Autoantibodies	13 (29.5)	8 (10.7)	.073
Abnormal thyroid function	31 (70.5)	43 (57.3)	.309
Presence of teratoma	2 (4.5)	1 (1.3)	.554
Abnormal EEG	37 (84.1)	64 (85.3)	.855
Abnormal brain MRI	9 (20.5)	14 (18.7)	.812

Abbreviations: CSF, cerebrospinal fluid; EEG, electroencephalography; IQR, interquartile range; LPP, lumbar puncture pressure; mRS, modified Rankin scale; MRI, magnetic resonance imaging; OFLD, orofacial dyskinesias; TOA, time from onset to admission; TOD, time from onset to diagnosis.

### 3.3 Associations of demographic information, clinical characteristics, laboratory tests, and imaging features with OFLD

Increased mRS at admission (OR, 1.82; 95% CI, 1.26–2.63; *p *= .002), serum sodium (OR, 1.12; 95% CI, 1.00–1.25; *p *= .035), and LPP (OR, 1.01; 95% CI, 1.00–1.01; *p *= .026) were associated with OFLD in univariate logistic regression model. Univariate logistic regression analysis also showed that the female biologic sex (OR, 5.23; 95% CI, 2.20–12.40; *p *< .001), fever (OR, 5.73; 95% CI, 2.35–13.97; *p *< .001), psychiatric symptoms (OR, 4.71; 95% CI, 1.51–14.66; *p *= .008), seizures (OR, 3.15; 95% CI, 1.24–8.01; *p *= .016), impaired consciousness (OR, 2.77; 95% CI, 1.21–6.32; *p *= .016), autonomic dysfunction (OR, 6.91; 95% CI, 1.37–34.91; *p *= .019), and central hypoventilation (OR, 4.67; 95% CI, 1.50–14.52; *p *= .008) were associated with OFLD. Multivariate regression analysis revealed that the female biologic sex (OR, 4.73; 95% CI, 1.27–17.64; *p *= .021), increased mRS at admission (OR, 2.09; 95% CI, 1.18–3.71; *p *= .011), psychiatric symptoms (OR, 7.27; 95% CI, 1.20–43.91; *p *= .031), and seizures (OR, 5.11; 95% CI, 1.22–21.43; *p *= .026) were associated with OFLD after adjusting for confounding factors (variables with *p* < .1 in univariate logistic regression model), including age, female biologic sex, length of stay, mRS at admission, fever, psychiatric symptoms, seizures, impaired consciousness, autonomic dysfunction, central hypoventilation, sleep disturbance, serum sodium, LPP, CSF protein, and autoantibodies. There were no significant associations between other clinical features or laboratory tests and OFLD (all *p* > .05). Table 2 presents the results of logistic regression analysis.

**TABLE 2:** Logistic regression for clinical factors of orofacial dyskinesias (OFLD)

	OFLD					
	Univariable logistic regression			Multivariable logistic regression^a^		
	OR	95% CI	*p*‐Value	OR	95% CI	*p*‐Value
**Demographic and clinical characteristics**						
Age, years	0.97	0.95–1.00	.057	–	–	–
Sex, female	5.23	2.20–12.40	<.001	4.73	1.27–17.64	.021
Length of stay, days	1.01	1.00–1.03	.098	–	–	–
TOA (range), days	0.99	0.98–1.01	.354	–	–	–
TOD (range), days	0.99	0.97–1.02	.450	–	–	–
mRS at admission	1.82	1.26–2.63	.002	2.09	1.18–3.71	.011
Headache	0.78	0.36–1.72	.544	–	–	–
Fever	5.73	2.35–13.97	<.001	–	–	–
Cognitive decline	1.18	0.52–2.67	.699	–	–	–
Psychiatric symptoms	4.71	1.51–14.66	.008	7.27	1.20–43.91	.031
Seizures	3.15	1.24–8.01	.016	5.11	1.22–21.43	.026
Impaired consciousness	2.77	1.21–6.32	.016	–	–	–
Autonomic dysfunction	6.91	1.37–34.91	.019	–	–	–
Speech disturbance	1.64	0.72–3.71	.237	–	–	–
Central hypoventilation	4.67	1.50–14.52	.008			
Sleep disturbance	2.44	0.98–6.06	.055			
**Laboratory tests, EEG and brain MRI**						
Blood leukocyte, 10^9^/L	0.98	0.89–1.08	.693	–	–	–
Serum sodium, 10^9^/L	1.12	1.01–1.25	.035	–	–	–
Serum potassium, 10^9^/L	0.71	0.26–1.96	.512	–	–	–
Serum calcium, 10^9^/L	1.98	0.23–16.86	.532	–	–	–
LPP, mmHg	1.01	1.00–1.01	.026	–	–	–
CSF cell count, per/μl	1.00	1.00–1.01	.402			
CSF protein, g/L	0.26	0.05–1.26	.094			
Tumor biomarkers	1.10	0.48–2.49	.829			
Rheumatic indicators	1.18	0.38–3.65	.775			
Autoantibodies	2.51	0.90–6.95	.077			
Abnormal thyroid function	1.60	0.64–3.99	.311			
Presence of teratoma	3.52	0.31–40.03	.310			
Abnormal EEG	0.91	0.32–2.55	.855			
Abnormal brain MRI	1.12	0.44–2.85	.812			

Abbreviations: CSF, cerebrospinal fluid; CI, confidence interval; EEG, electroencephalography; LPP, lumbar puncture pressure; mRS, modified Rankin scale; MRI, magnetic resonance imaging; OR, odds ratios; TOA, time from onset to admission; TOD, time from onset to diagnosis.

^a^ Adjusted for confounding factors including age, female biologic sex, length of stay, mRS at admission, fever, psychiatric symptoms, seizures, impaired consciousness, autonomic dysfunction, central hypoventilation, sleep disturbance, serum sodium, LPP, CSF protein, and autoantibodies.

## 4 DISCUSSION

This study investigated the relationship between clinical factors and OFLD in anti‐NMDAR encephalitis. The results showed that clinical factors, such as the female biologic sex, increased mRS at admission, psychiatric symptoms, and seizures, were independently associated with OFLD. Thus, our findings suggest that these clinical factors of anti‐NMDAR encephalitis are associated with a high risk of developing OFLD.

The female biologic sex was independently associated with OFLD in the present study. Our findings are consistent with data from previous studies. Specifically, Varley et al. (2019) reported that individuals with a female biologic sex were more likely to have MDs and anti‐NMDAR encephalitis occurring co‐morbidly. However, the sex‐related differences in MDs are insufficiently studied and poorly understood (Meoni et al., 2020). Sex hormones in women, such as estrogens, may justify some differences, implicating their crucial role in the MDs (Rabin et al., 2014). An underlying explanation could account for this association: estrogen may aggravate hyperkinetic states (such as chorea) (Meoni et al., 2020). The pathophysiological mechanisms of sex‐related differences in MDs should be investigated in future studies.

Symptom severity was assessed with the mRS at admission of the disease in the present study. We found that increased mRS at admission was independently associated with OFLD in patients with anti‐NMDAR encephalitis. There is growing evidence that mRS can be used to evaluate neurological outcomes in anti‐NMDAR encephalitis (Titulaer et al., 2013; Xu et al., 2019). However, the relationship between the mRS and OFLD has not been reported previously to the best of our knowledge. Therefore, our findings indicated that it is valuable to assess the neurological status at admission.

We observed that psychiatric symptoms are significantly associated with OFLD. It is widely accepted that both psychiatric symptoms and MDs are predominant manifestations in anti‐NMDAR encephalitis (Dalmau et al., 2017; Irani et al., 2010). Although the relationship between psychiatric symptoms and OFLD was not clear, most previous studies proposed that abnormalities in glutamatergic receptor functions play a crucial role in the pathogenesis of psychosis and MDs (Iizuka et al., 2008; Laruelle, 2014). Therefore, we speculate that this neurobiological phenomenon may explain why these two conditions coexist clinically in anti‐NMDAR encephalitis. However, further prospective studies are required to elucidate the underlying mechanism of the relationship.

A significant association was found between seizures and OFLD after adjusting for confounding factors in the present study. Although clinical manifestations of seizures and MDs are different, multiple lines of evidence show that both conditions could coexist and overlap in patients with anti‐NMDAR encephalitis (Freitas et al., 2019). For example, focal seizures with motor‐onset symptoms can be mistaken for different MDs, and automatisms in focal seizures may mimic OFLD. Traditionally, seizures have been attributed to disorders in the cerebral cortex, while MDs have mostly been reported to reflect dysfunction in subcortical areas (Freitas et al., 2019). However, such a dichotomous distinction between subcortical and cortical events is likely an oversimplification due to diffuse and complex connections between the basal ganglia and cerebral cortex, especially the frontostriatal pathway (Freitas et al., 2019). For example, in a case of concurrent seizures and MDs, the epileptogenic source was determined in the motor cortex, while the ictal discharge was also detected in the basal ganglia (van Strien et al., 2012). Hence, both the cerebral cortex and basal ganglia might be attacked by anti‐NMDAR antibodies, leading to MDs and seizures. However, further prospective studies are still necessary to confirm this pathogenesis.

The present study had several limitations. First, this study was retrospective, and a longitudinal study is necessary in the future. Second, our study was a single center study. Thus, our results are not generalizable. Future research should include multicenter trials with larger sample sizes. Finally, we only confirmed the association of demographic and clinical characteristics with OFLD in patients with anti‐NMDAR encephalitis. OFLD is a distinctive feature of NMDAR encephalitis (Dalmau et al., 2008; Irani et al., 2010), while another abnormal movement is required for further subgroup analysis in future prospective studies.

## 5 CONCLUSION

The present study showed that clinical factors, such as female biologic sex, increased mRS at admission, psychiatric symptoms, and seizures, were independently associated with OFLD. Our findings suggest that it is valuable to evaluate the demographic and clinical characteristics to characterize the risk of OFLD and may even warrant more closely monitored symptom progression and development.

## CONFLICT OF INTEREST

The authors declare no conflict of interest.

## AUTHOR CONTRIBUTIONS

*Conceptualization (lead), investigation (lead), formal analysis (lead), writing‐original draft (equal), and writing‐review and editing (equal)*: Hailun Hang. *Data curation (equal), writing‐original draft (equal), and writing‐review and editing (equal)*: Liuyu Lin. *Data curation (equal), writing‐original draft (equal), and writing‐review and editing (equal)*: Danhui Li. *Data curation (equal), writing‐original draft (equal), and writing‐review and editing (equal)*: Jin Li. *Data curation (equal), writing‐original draft (equal), and writing‐review and editing (equal)*: Jingping Shi. *Data curation (equal), supervision (lead), writing‐original draft (equal), writing‐review and editing (equal), and funding acquisition (lead)*: Jie Lu.

### PEER REVIEW

The peer review history for this article is available at https://publons.com/publon/10.1002/brb3.2638.

## Data Availability

The data that support the findings of this study are available from the corresponding author upon reasonable request.
